# Effectiveness and safety of self-pulling and latter transected reconstruction in totally laparoscopic total gastrectomy: a comparison with laparoscopic-assisted total gastrectomy

**DOI:** 10.1186/s12893-023-02077-5

**Published:** 2023-06-29

**Authors:** Defei Chen, Fuyu Yang, Fan He, Saed Woraikat, Chenglin Tang, Kun Qian

**Affiliations:** Department of Gastrointestinal Surgery, The First Affiliated Hospital of Chongqing Medical University, Chongqing, 400016 China

**Keywords:** Totally laparoscopic, Self-pulling and latter transection, Total gastrectomy

## Abstract

**Background:**

In some earlier studies, self-pulling and later transection (SPLT) esophagojejunostomy (E-J) was incorporated into total laparoscopic total gastrectomy (TLTG) procedures. Its effectiveness and safety, however, remain unknown. This study compared (SPLT)-E-J in TLTG with conventional E-J in laparoscopic-assisted total gastrectomy (LATG) in order to assess the short-term safety and efficacy of (SPLT)-E-J in TLTG.

**Methods:**

This research analyzed patients with gastric cancer who received SPLT-TLTG or LATG between January 2019 and December 2021 in the First Affiliated Hospital of Chongqing Medical University. Baseline data and postoperative short-term surgical outcomes were obtained retrospectively and compared between the two groups.

**Results:**

A total of 83 patients who underwent SPLT-TLTG (n = 40, 48.2%) or LATG (n = 43, 51.8%) were included in this study. There were no differences between the two groups in terms of patient demographics or tumor characteristics. No statistically significant differences were observed between the two groups in terms of operation time, intraoperative blood loss, harvested lymph nodes, postoperative complications, postoperative decrease in hemoglobin and albumin levels, or postoperative hospital stay. Five and seven patients experienced short-term postoperative complications in the SPLT-TLTG and LATG groups, respectively.

**Conclusions:**

SPLT-TLTG is a dependable and safe surgical method for the treatment of gastric cancer. Its short-term outcomes were similar to those of conventional E-J in LATG and had advantages regarding surgical incision and simplification of reconstruction.

## Introduction

Globally, gastric cancer is one of the most prevalent malignant tumors of the digestive tract, and its morbidity and mortality among malignant tumors rank fourth and fifth, respectively [1]. Asian has one of the highest gastric cancer incidences [2]. Currently, the treatment strategy for gastric cancer is a comprehensive treatment based on radical gastrectomy. Laparoscopic gastrectomy has the advantages of a better visual field, lesser incisions, and quicker postoperative recovery than open gastrectomy [3].

Total laparoscopic gastrectomy (TLG) is a radical gastrectomy for gastric cancer in which all surgical steps, including lymph node dissection, gastrectomy, and gastrointestinal reconstruction, are performed laparoscopically [4]. In the past 30 years, with the development of laparoscopic instruments and the improvement of surgical techniques, particularly the development of linear staplers and the application of delta-shaped anastomosis, TLG has been widely used in distal gastrectomy [5–8]. However, because the operational techniques for performing esophagojejunostomy (E-J) under laparoscopic surgery are complex, totally laparoscopic total gastrectomy (TLTG) remains challenging and unpopular, although it has been verified as safe and feasible [8]. Compared with laparoscopic-assisted total gastrectomy (LATG), when performing TLTG, reconstruction is completed under laparoscopy so that purse-string sutures and placing a nail anvil through an auxiliary incision are avoided, which contributes to a better reconstruction view and less trauma, especially in patients with obesity or those with narrower costal arches [9, 10].

- E-J is the most challenging part of TLTG, especially for tumors invading the lower esophagus, significantly the main reason TLTG is not generally used. There are many methods for performing E-J in TLTG. The main methods using circular staplers include the oral anvil insertion system (OrVil™) [11, 12] and the reverse puncture method [13, 14]. In OrVil™, the anvil is inserted through a transoral passage, and because the anvil can’t be seen when it was pulled through the esophagus, there is a risk of damage the esophagus. For the reverse puncture method, the anvil is inserted through a small incision on the side of the esophageal stump, and subsequently, the tip of the anvil is pulled out from the side of the esophagus 1–2 cm above the small incision with a thread or tube while the esophagus is transected with a linear stapler. The main anastomosis methods using linear staplers include functional end-to-end anastomosis (FEEA) [15] and overlapping side-to-side anastomosis (Overlap) [16]. Differing from antiperistaltic anastomosis in FEEA, Overlap is a peristaltic anastomosis, so it can improve the anastomotic corner problem in FEEA [17] and is superior to other types of anastomosis in reducing anastomotic stenosis rates [18]. Compared with tubular anastomosis, linear anastomosis has a lower risk of postoperative anastomotic stenosis [19], and it can be completed through the trocar rather than through an additional small incision, which is necessary in tubular anastomosis. However, it is still difficult to complete laparoscopic E-J after gastrectomy because of the retraction of the esophagus and occlusion of the diaphragm after total gastrectomy.

Consequently, methods based on FEEA and Overlap have been proposed, among which the most famous are self-pulling and transected-E-J (SPLT)-E-J [20] and π-type anastomosis [21]. When performing SPLT-E-J, E-J is completed before cutting off the esophagus and jejunum, which reduces the difficulty of the operation. However, there are few studies on the safety and feasibility of this procedure. Therefore, this retrospective study aimed to evaluate the short-term safety and efficacy of SPLT-E-J in TLTG at our center by comparing it with conventional E-J in LATG.

## Materials and methods

### Patients

SPLT-TLTG was launched as a treatment option at our institution in January 2019 for patients who fulfilled the following inclusion criteria: (1) gastric adenocarcinoma confirmed by pathological biopsy; (2) contrast-enhanced computed tomography (CT) performed prior to surgery to confirm T1–4a; (3) no distant metastasis (M0); and (4) tumor located in the gastric body, fundus, entire stomach, or cardia, and if there was any invasion of intra-abdominal esophagus, no more than 2 cm above the cardia.

**Fig. 1:**
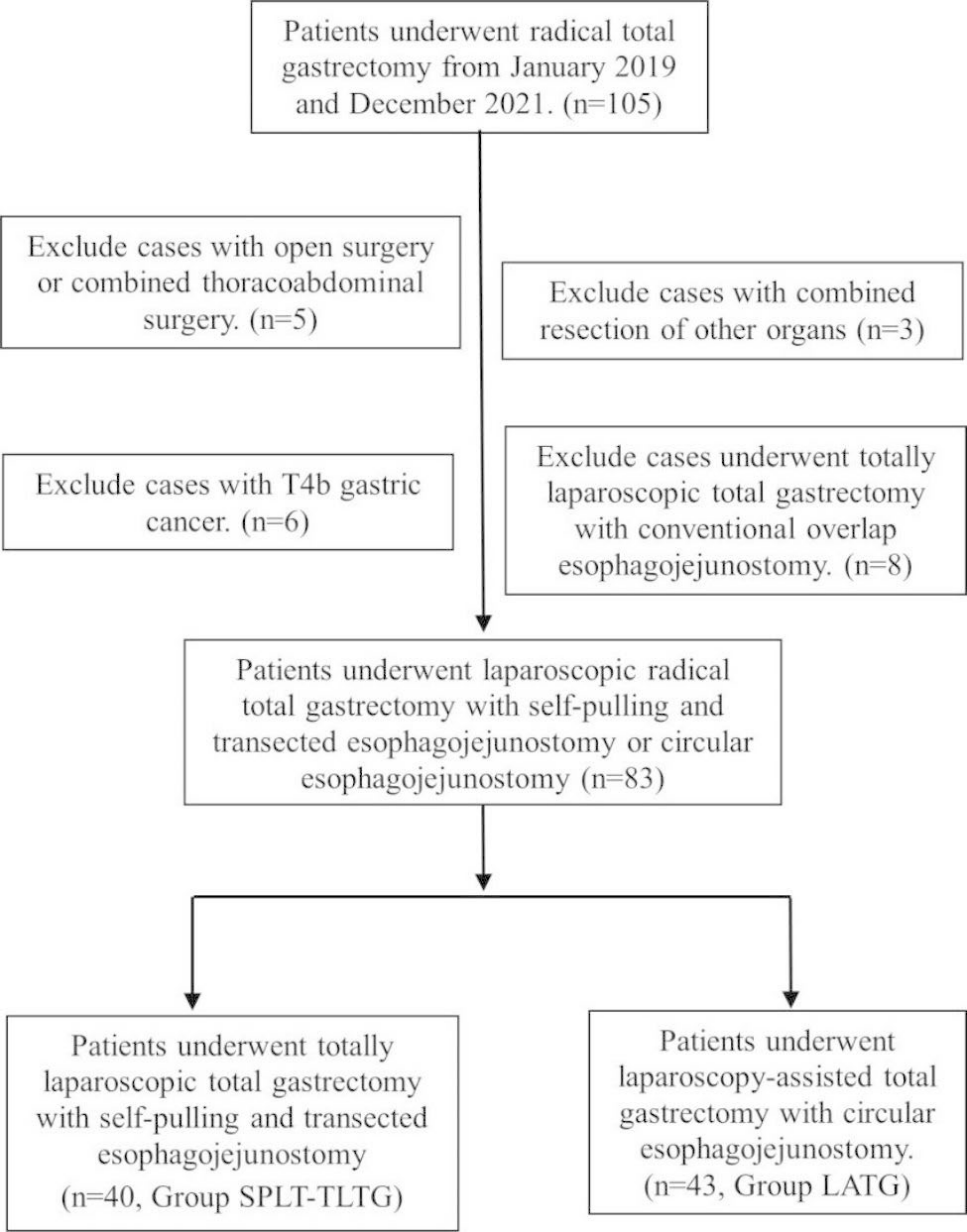
Enrollment of patients in the study Abbreviations: LATG, laparoscopy-assisted total gastrectomy; SPLT, self-pulling and later transected; TLTG, totally laparoscopic total gastrectomy

Patients were informed about the positive and negative aspects of the two surgical treatments (SPLT-TLTG or LATG) through a preoperative interview and then selected the surgical method by signing an informed consent form. All the procedures were performed by the same surgeon who had more than 10 years of experience in gastrointestinal surgery and had performed over 100 laparoscopic radical gastric procedures. Between January 2019 and December 2021, 105 patients underwent laparoscopic radical total gastrectomy in our center. Of these patients, 5 patients with open surgery or combined thoracoabdominal surgery, 3 patients with combined resection of other organs, 6 patients with T4b gastric cancer, and 8 patients who underwent TLDG with overlap esophagojejunostomy were excluded. The remaining 83 patients were enrolled in the study. SPLT-TLTG was performed in 40 patients (Group SPLT-TLTG), and LATG with a circular anastomosis was performed in 43 patients (Group LATG) (Fig. 1). Data from patients in two groups were collected and analyzed objectively.

### Procedures of SPLT-TLTG

The patient was positioned in a supine posture with spread legs following general anesthesia. The surgeon stood on the patient’s left side, the assistant stood on the patient’s right side, and the camera operators stood between the patient’s legs.

**Fig. 2:**
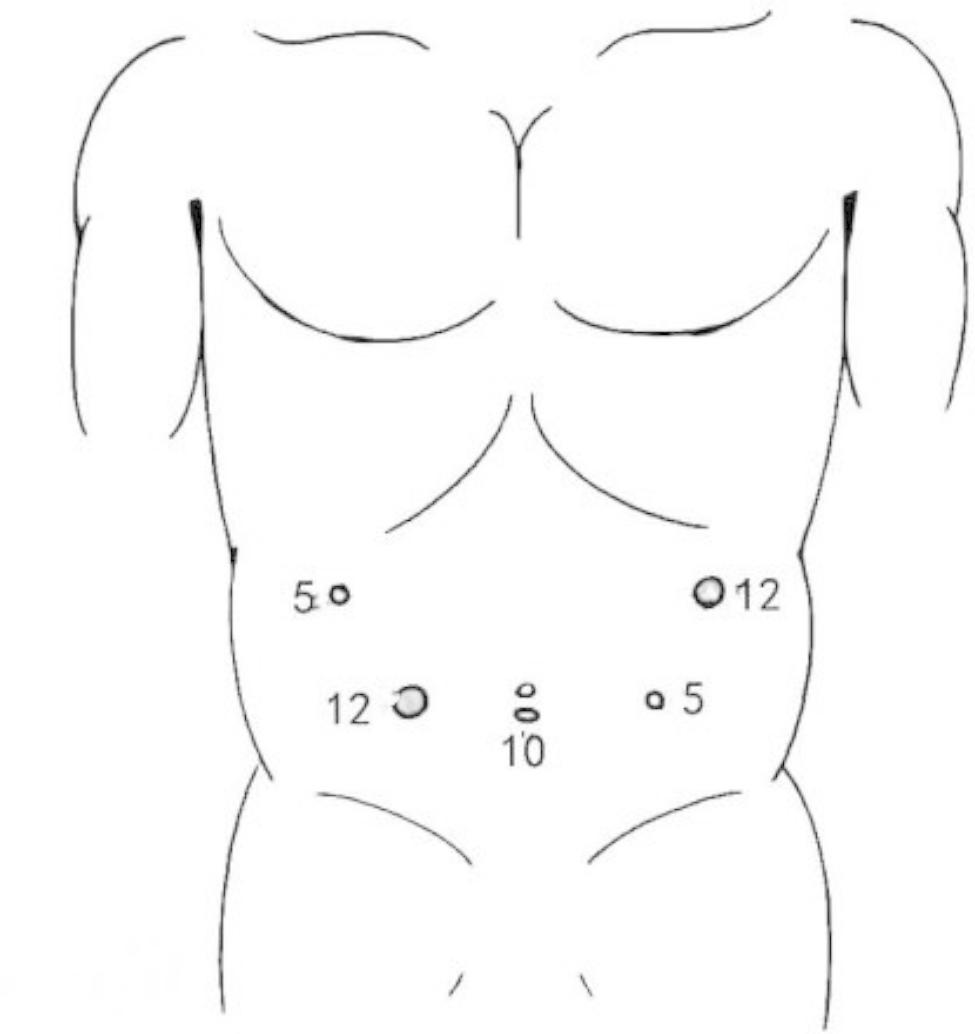
Placement of the trocars

Moreover, a pneumoperitoneum with a pressure of 1.6 kPa was routinely established. Following the placement of three trocars for laparoscopic investigation, two more trocars were placed once the tumor’s location and the absence of any metastases were confirmed. The five trocars were positioned as follows: (1) a 10-mm trocar was inserted 1 cm below the umbilicus; (2) a 12-mm and a 5-mm trocar were inserted 2 cm below the lower border of the costal arch at the left, and right anterior axillary lines; (3) and a 12-mm and a 5-mm trocar were inserted on both sides of the lower quadrant on the umbilicus line (Fig. 2).

Subsequently, following the completion of D2 lymph node dissection in accordance with the Japanese Gastric Cancer Association guidelines [22], the SPLT-E-J reconstruction performed refers to the procedures introduced by Hao et al. [20] and is in line with the subsequent procedures: (1) the duodenum was cut off at the duodenal bulb; (2) the lower esophagus was ligated with sterile twine; (3) the esophagus was pulled to the left upper abdomen; (4) a hole was punched in the right posterior wall of the esophagus 3 cm above the ligature line; (5) another hole was punched in the small intestine on the opposite side of the mesentery, located approximately 20 cm from the Treitz ligament; (6) the two staple plates from the linear stapler were inserted into the two holes, the jejunum and esophagus were buttressed, and the lateral E-J was completed after inspection; (7) the proximal esophagus and jejunum were transected, and the common opening was simultaneously closed with a linear stapler; (8) holes were punched at the anterior margin of the small intestine 40 cm from the E-J anastomosis and 1 cm from the proximal jejunal margin, respectively; (9) the previous holes were used to complete the side-to-side jejunojejunostomy (J-J) and then the common opening was closed with linear staplers; (10) the specimen was placed in a specimen bag and then a 3–4-cm transverse abdominal incision was made in the lower abdomen and the specimen was finally removed; (11) A nasogastric tube was placed about 20 cm below the E-J anastomosis. and absence of bleeding at each anastomosis was verified; (12) the instruments were checked, the incision was closed, and the procedure was ended.

### Procedures of LATG

All steps prior to reconstruction in LATG were the same as those used in SPLT-TLTG. Following the D2 LN dissection, the duodenum was cut off from the duodenal bulb, and the pneumoperitoneum was released. A 7–8-cm incision protected by an incision protector was made in the exact center of the epigastrium. The stomach was then pulled out, the lower end of the esophagus was secured with purse-string forceps, and the stomach was cut off with a linear cutting stapler and removed alongside the previously dissected tissue. After completing the purse-string suture, the purse-string forceps were removed, the anvil was placed into the purse, and lastly, the purse string was tightened. The mesentery was then dissected, and a hole was made in the jejunum approximately 20 cm from the Treitz ligament. A circular stapler was then used to conduct end-to-side E-J, and a linear stapler was subsequently utilized to seal the opening. A J-J anastomosis was performed using two linear staplers after a hole was cut at the antimesenteric boundary of the small intestine at a 45-cm distance from the E-J anastomosis. A nasogastric tube was placed about 20 cm below the E-J anastomosis and all anastomoses and stumps were examined for bleeding, the incision was closed, and the surgery was complete.

### Postoperative management

**Fig. 3:**
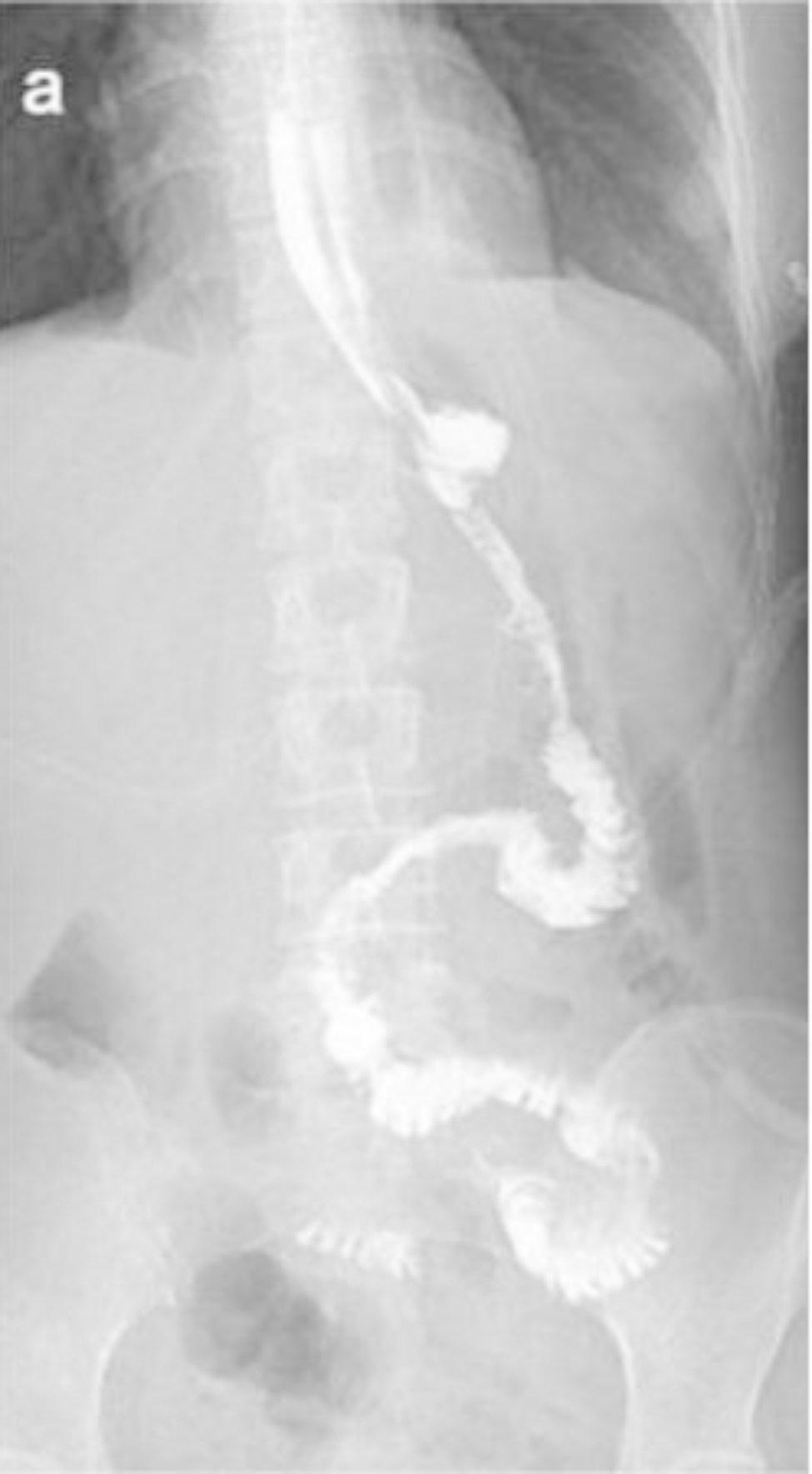
Upper gastrointestinal angiography using iohexol on day 3 after SPLT-TLTG. The anastomotic stoma was unobstructed, with no contrast agent overflow and no anastomotic stenosis, and the distal jejunum was unobstructed Abbreviations: SPLT, self-pulling and later transected; TLTG, totally laparoscopic total gastrectomy

All patients in the research received standard postoperative treatment, including broad-spectrum antibiotics for 48 h after surgery and octreotide and proton pump inhibitors until liquid intake. Patients were advised to walk around on the first postoperative day. Nasogastric tube was removed 2 days after the procedure, unless the drainage character was abnormal. Upper gastrointestinal water-soluble contrast radiography was typically performed for 3 days after the procedure (Fig. 3). A liquid diet was recommended if no anastomotic leakage was observed on upper gastrointestinal water-soluble contrast radiography or if the patient had experienced flatus or had a bowel movement. As their bowel movements returned to normal and they indicated no discomfort from the liquid diet, patients without complications were discharged. Anastomotic stenosis was defined as a condition that required endoscopic dilatation. Anastomotic leakage was evaluated and proven by water-soluble contrast radiography. A duodenal stump fistula was diagnosed when fluid containing bile was collected from the indwelling drainage tube placed beside the duodenal stump. Pancreatic fistula was described according to the definition proposed by the International Study Group on Pancreatic Fistula [23]. An abdominal cavity infection was diagnosed when a purulent culture-positive specimen was obtained from the indwelling drainage tube.

### Data collection

Preoperative, intraoperative, and postoperative data on all the research participants were gathered as baseline data. Sex, body mass index (BMI), preoperative hemoglobin level, preoperative albumin level, tumor stage, and significant medical history were all included in the preoperative data. Operative time, intraoperative blood loss, blood transfusion, and harvested lymph nodes were included in the intraoperative data. Time of ambulation, first flatus, and first fluid intake, postoperative hospital stay, decrease in hemoglobin and albumin levels, and complications such as anastomotic leakage, duodenal stump leakage, abdominal infection, anastomotic stenosis, esophagitis, postoperative bleeding, incision, and pulmonary infection were all recorded in postoperative data.

### Statistical analysis

All statistical analyses were performed using SPSS software (version 26.0; IBM Inc., Armonk, NY, USA). Differences in continuous variables between the two groups were tested using the Mann–Whitney U test. Differences in ordered categorical variables were compared using the chi-squared or Fisher’s exact tests. Statistical significance was set at *P* < 0.05.

## Results

Overall, 83 patients were included in this study, and the baseline information of the two groups was compared (Table 1). No significant between-group differences were observed in sex, BMI, ASA scores, preoperative hemoglobin and albumin levels, tumor characteristics, or medical history, including abdominal surgery history.

**Table 1:** Patient demographics and tumor characteristics of both groups

Characteristics	SPLT-TLTG (*n* = 40)	LATG (*n* = 43)	*P* value
Age (years) ^a^	60.30 ± 11.80	57.95 ± 14.02	0.64
Sex (Male/Female) ^b^	31/9	31/12	0.57
BMI (kg/m^2^ ) ^a^	22.01 ± 2.42	22.32 ± 3.64	0.62
Smoking ^b^	20(50%)	20(46.5%)	0.75
Drinking ^b^	15(37.5%)	15(34.9%)	0.80
Abdominal surgery history ^b^	7(17.5%)	6(14.0%)	0.66
Main comorbidity Hypertension/T2DM/COPD ^b^	2/1/4	6/5/0	0.37
ASA score (1/2/3/4) ^b^	8/22/10	10/21/12	0.85
Preoperative hemoglobin (g/L) ^a^	117.18 ± 22.28	114.60 ± 25.88	0.63
Preoperative albumin (g/L) ^a^	39.75 ± 4.70	38.40 ± 6.02	0.14
Neoadjuvant chemotherapy ^b^	13(32.5%)	14(32.6%)	1.00
The upper boundary of the tumor Cardia/fundus/body ^b^	15/5/20	16/7/20	0.88
Tumor size (cm) ^a^	3.46 ± 1.94	3.34 ± 2.15	0.61
TNM stage ^b^	1/12/11/16	3/8/13/19	0.56
Degree of differentiation			
(low/medium/high) ^b^	25/15/0	30/12/1	0.48

^a^Data are presented as mean ± standard deviation. ^b^Data are presented as n corresponding to groups

T2DM, type 2 diabetes mellitus; COPD, chronic obstructive pulmonary disease; BMI, body mass index; ASA, American Society of Anesthesiologists; SPLT, self-pulling and later transected; TLTG, totally laparoscopic total gastrectomy; LATG, laparoscopy-assisted total gastrectomy

Table 2 shows the operative and postoperative patient data. All 83 patients successfully underwent either SPLT-TLTG (n = 40, 48.2%) or LATG (n = 43, 51.8%). None of the patients underwent open surgery, and intracorporeal anastomosis was successfully performed in all the patients in the SPLT-TLTG group. The mean operation time was similar between the SPLT-TLTG and LATG groups. No significant differences were found in intraoperative blood loss, number of LNs harvested, time to ambulation, time to first flatus, time to first liquid intake, length of postoperative hospital stay, and decrease in hemoglobin and albumin levels between the two groups.

**Table 2:** Comparison of surgical outcomes between SPLT-TLTG and LATG

Characteristics	SPLT-TLTG(*n* = 40)	LATG(*n* = 43)	*P* value
Operation time (min)	195.38 ± 60.60	182.16 ± 46.60	0.43
Intraoperative blood loss (mL)	113.63 ± 122.97	152.09 ± 390.81	0.33
Harvested lymph nodes	23.88 ± 8.10	22.86 ± 9.13	0.40
Time to ambulation (days)	1.43 ± 0.84	1.35 ± 0.61	0.93
Time to first flatus (days)	2.75 ± 1.58	2.91 ± 1.38	0.41
Time to first liquid intake (days)	6.50 ± 4.14	7.47 ± 4.96	0.21
Postoperative hospital stay (days)	11.50 ± 6.13	13.72 ± 10.87	0.51
Decrease in hemoglobin (g/L)	4.18 ± 10.07	7.12 ± 16.66	0.56
Decrease in albumin (g/L)	8.32 ± 4.34	7.33 ± 5.77	0.19
Intraoperative blood transfusion	3(7.5%)	4(9.3%)	1.00

Variables are expressed as mean ± standard deviation or n (%)

SPLT, self-pulling and later transected; TLTG, totally laparoscopic total gastrectomy; LATG, laparoscopy-assisted total gastrectomy

Table 3 shows the postoperative complications. Complications were graded according to the criteria proposed by Clavien and Dindo, and only those rated as grade > 2 were recorded [24]. In the SPLT-TLTG group, 5 (12.5%) patients experienced postoperative complications, which were not significantly different from the 7 (16.3%) patients in the LATG group (*P* = 0.625). Additionally, 2 and 3 patients in the SPLT-TLTG and LATG groups, respectively, had E-J anastomotic leakage, all of whom were diagnosed by upper gastrointestinal angiography. Among the five patients who experienced E-J anastomotic leakage, two patients were found to have pleural effusion during angiography, and thoracentesis was needed by one of them. Nasojejunal feeding tube placement was performed in four patients, and all five patients recovered and were discharged after anti-infection, drainage, and nutritional support treatment. One patient in the LATG group developed anastomotic stenosis 1 month after the operation, and the symptoms were relieved after two gastroscopic balloon dilations of the anastomotic stoma. One patient in the SPLT-TLTG group experienced postoperative ileus, which was cured by fasting and gastrointestinal decompression. In addition, one pancreatic fistula in the SPLT-TLTG and LATG groups, one abdominal infection in the SPLT-TLTG group, one duodenal stump fistula, and pulmonary infection in the LATG group all recovered and were discharged after drainage and anti-infection treatment. No second surgery or hospital death occurred in either group. During the follow-up period of 6 months at least, none of the patients complained of reflux symptoms or experienced tumor recurrence or metastasis.

**Table 3:** Comparison of postoperative complications between SPLT-TLTG and LATG

Characteristics	SPLT-TLTG(*n* = 40)	LATG(*n* = 43)	*P* value
No. of postoperative complications	5(12.5%)	7(16.3%)	0.63
Type			
Duodenal stump fistula	0	1	
Anastomotic leakage	2	3	
Anastomotic stenosis	0	1	
Postoperative bleeding	0	0	
Pancreatic fistula	1	1	
Postoperative Ileus	1	0	
Abdominal cavity infection	1	0	
Pulmonary infection	0	1	
Dindo–Clavien grade			
II/IIIa/IIIb/IV	3/2/0/0	3/3/1/0	1.00
Requiring of reoperation	0	0	
Readmission	0	0	
Hospital death	0	0	

Variables are expressed as n (%)

SPLT, self-pulling and later transected; TLTG, totally laparoscopic total gastrectomy; LATG, laparoscopy-assisted total gastrectomy

**Fig. 4:**
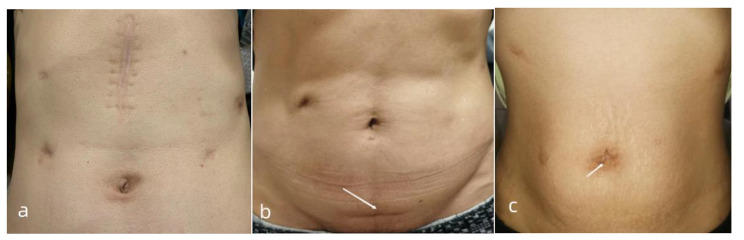
Abdominal photograph 6 months after surgery. (**a**) Abdominal photograph 6 months after LATG; (**b**) Abdominal photograph 6 months after SPLT-TLTG, the surgical specimen was taken out through the lower abdominal transverse incision (indicated by the arrow); (**c**) Abdominal photograph 6 months after SPLT-TLTG, the surgical specimen was taken out through the extended trocar hole below the umbilicus (indicated by the arrow) Abbreviations: LATG, laparoscopy-assisted total gastrectomy; SPLT, self-pulling and later transected; TLTG, totally laparoscopic total gastrectomy

## Discussion

Recently, laparoscopic gastrectomy has become the mainstream procedure for gastric cancer treatment with the rapid development of laparoscopic instruments and surgeons’ surgical skills. Conventional laparoscopic gastrectomy requires auxiliary incisions (Fig. 4a) to complete reconstruction after gastrectomy, which is not totally laparoscopic gastrectomy. During total laparoscopic gastrectomy, all surgical procedures, including LN dissection, gastrectomy, and reconstruction, were completed under laparoscopic vision. After reconstruction was completed, the specimens were removed through a small incision. Compared with LATG, the surgical incision of TLTG is smaller, and the choice of incision site is also more flexible. Additionally, the specimen can be taken out through the lower abdominal transverse incision (Fig. 4b) or can be removed through the extended trocar hole below the umbilicus (Fig. 4c) or from the natural orifice, including the rectum or the female vagina, which contributes to more minimal invasion. In addition, TLTG does not need to drag the digestive tract out of the body for reconstruction, which reduces the dependence on surgical incisions and contributes to less trauma, lower risk of infection, and faster postoperative recovery. Moreover, in TLTG, the visual field of reconstruction is clearer, which enhances the security of anastomotic stoma [9, 10]. However, laparoscopic skills and experience are more important when performing TLTG.

The conventional laparoscopic-assisted E-J is primarily completed with purse-string forceps and circular staplers. Therefore, the initial TLTG also focused on the circular anastomosis, of which the most commonly used is the Orvil™ method [11, 12] and reverse puncture method [13, 14], both of which require the insertion of the anvil, which is difficult to complete. Compared with circular anastomosis, linear anastomosis has many advantages, including fewer requirements on the diameter of the digestive tract, a lower probability of anastomotic stenosis, and the stapler can be placed through the 12 mm trocar hole, which is widely used in TLTG [5–8]. In TLTG, the main methods using linear staplers are the FEEA method [15] and the overlap method [16]. Compared with reversed peristalsis anastomosis in FEEA, the overlap is a straight-forward anastomosis, which can dismiss the corner problem existing in the FEEA and effectively improve the safety of the anastomosis [17]. The anastomosis of these two methods is completed after gastrectomy, and releasing the diaphragm angle in advance is often necessary because of the esophagus retraction after the stomach is severed and the occlusion of the diaphragm. However, the alignment of the esophagus and jejunum is still challenging during the anastomosis, especially when the tumor invades the lower esophagus; the visual field of the anastomosis is worse the safety of the anastomosis cannot be guaranteed. In SPLT-TLTG, a rope was used to ligate the lower esophagus for self-traction. E-J was conducted first, and then the esophagus and jejunum were separated, which greatly reduced the difficulty of the E-J procedure. Nevertheless, similar to the π-type anastomosis, in SPLT-TLTG, the stomach specimen can only be obtained at the end of the reconstruction. Therefore, when the safety of the resection margin is questionable, the choice of the anastomosis method should be cautious, which means that preoperative and intraoperative assessment of the tumor location is crucial [20, 21]. In addition, because pulling on the esophagus can easily form a false tract between the esophageal adventitia and esophagus, it is necessary to ensure that the staple plate of the stapler is located in the esophagus by moving the gastric tube during the anastomosis; otherwise, the consequences are disastrous.

In a previous study, compared with LATG, TLTG was proven to have the advantages of reduced intraoperative blood loss, a greater number of retrieved lymphatic nodes, decreased hospitalization duration, reduced incision length, and shorter time to first fluid diet [9]. In addition to being more minimally invasive in this study, the advantages of SPLT-TLTG compared to LATG were not apparent, which may be due to the small sample size of this study. Additionally, compared with the study of Hao et al. [20, 25], the operation time in this study was shorter, which may be due to the differences in the surgeon’s surgical skill, experience, and the instruments used. In conclusion, combined with the results of this study and previous studies, compared with laparoscopic-assisted surgery, the advantages of SPLT-TLTG in terms of minimal invasiveness and reduced surgical difficulty are evident.

This study had some limitations. First, this was a retrospective study with small sample size. Second, it lacked relevant data on long-term quality of life and survival after surgery. In addition, although the surgeons in this study were experienced and skillful in laparoscopic surgery and had a short learning curve to perform new procedures, the impact of the learning curve on the study results could not be ignored. Therefore, the safety and feasibility of SPLT-TLTG still need to be evaluated in a large-scale, high-quality, randomized controlled trial.

## Conclusion

SPLT-TLTG is a safe and reliable surgical procedure for the treatment of gastric cancer. Its short-term outcomes were similar to those of conventional E-J in LATG and had advantages regarding surgical incision and simplification of reconstruction.

## Data Availability

The data sets used and analyzed during the current study are available from the corresponding author on reasonable request.
